# Human rights and global health emergencies preparedness

**DOI:** 10.7189/jogh.10.010334

**Published:** 2020-06

**Authors:** Andrea Boggio

**Affiliations:** Department of History and Social Sciences, Bryant University, Smithfield, Rhode Island, USA

**Figure:**
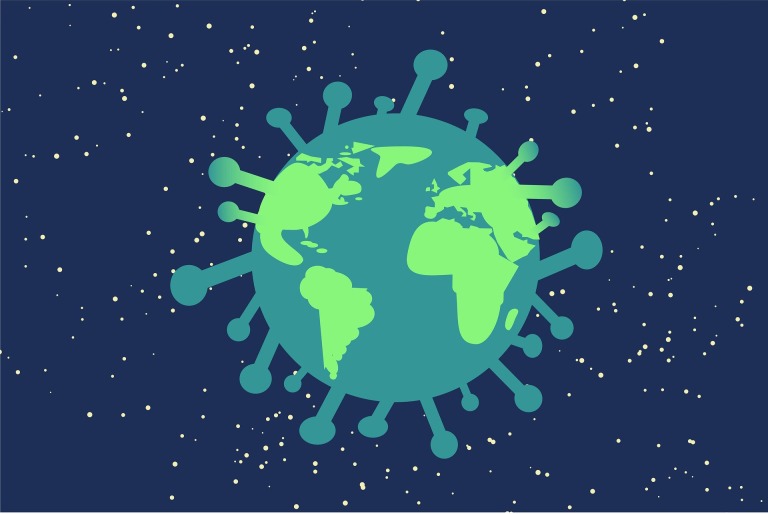
Photo: Image by Miroslava Chrienova from Pixabay.

The spread of infectious disease is always ranked high as a global threat. It features prominently among the list of urgent health challenges for the next decade, issued in early January 2020 by WHO [1]. The emergence of the 2019 Novel Coronavirus (COVID-19) in the Hubei Province in China, is a reminder of global health vulnerabilities.

Since the 2002-03 SARS outbreak, the global health community has engaged in substantial efforts to be prepared to handle outbreaks such as the ongoing COVID-19 outbreak. A milestone achievement is the International Health Regulations, a binding instrument of international law that entered into force on 15 June 2007 intending to assist countries to work together to save lives and livelihoods endangered by the international spread of diseases. The Regulations target the response to the international spread of disease as well as the core surveillance and response capacities of countries. Additionally, they give certain powers to State Parties (ie, closing ports, airports, and ground crossings) that governments cannot ordinarily exercise. Yet, these powers must be exercised with caution, in accordance with their relevant national law and obligations under international law, including human rights law, and upon considering scientific principles, available scientific evidence of a risk to human health, and WHO’s guidance or advice [2]. As Habibi and colleagues point out, the intention of the Regulations is that “countries should not take needless measures that harm people or that disincentivise countries from reporting new risks to international public health authorities” [3].

Yet, even when done correctly, in line with the scientific knowledge, and full respect of international human rights law, emergency handling and planning is not sufficient to manage the spread of infectious disease. To be effective, emergency handling and planning must be carried out in an environment in which new treatments – such as drugs and vaccines developed ad hoc to stop the outbreak – can be developed rapidly and delivered efficiently to all persons affected. This was not the case in the urban area of Wuhan, which suffered from health care delivery problems at the time of the outbreak. In a matter of days, the local authorities conclude that the health care facilities were insufficient to handle the emergency and announced plans to build from scratch not one but two new hospitals. These construction projects were completed in just a few days and accommodate 2300 patients. Similar problems are experienced by other nations as COVID-19 spreads.

This is extraordinary. But it should not be. Strengthening the health care infrastructure should have been planned for a while. The failure to do so is a cautionary tale of insufficient efforts to protect global health. The global health community must thus push countries harder to keep strengthening their research capacity and basic health care infrastructure along with emergency handling and planning efforts. Human rights play a key told in pushing countries hard. They are a powerful tool to act as they create legal obligations that go beyond the immediate and pressing needs of emergency preparedness and handling. International human rights law is critical as it transforms global health best practices in legal duties.

The leading treaty in this area is the Covenant on Economic, Social and Cultural Rights, which recognizes the right to health (Art. 12) and the right to science (Art. 15) [4]. While the right to health is often invoked in the global health arena – although only to a limited extent in the context of global health preparedness – the links between right to science and global health have received very little attention (with some exceptions in the area of drug-resistant TB policies) [5]. This provision imposes on State parties the duty to ensure that scientific knowledge is produced and translated into applications, such as drugs and vaccines, that are beneficial to rightsholders. For this, substantial public funds must be allocated on a regular basis to R&D. The Covenant also includes the duty to ensure a sufficient degree of scientific literacy in the population so that, when vaccines become available, patients readily embrace new treatments.

Human rights advocacy in this area is about to become more effective due to the recent approval by the UN Committee on Economic, Social and Cultural Rights of a general comment on the relationship between science and economic, social and cultural rights [6]. When the official version of the general comment is published (likely in April 202), this instrument will provide a clear framework of state obligations under article 15 of the Covenant. In fact, the draft that was made public makes the case. It stresses that governments have “a positive duty to actively promote the advancement of science” and must fund basic and applied research (para. 50). To this, it adopts the recommendation of the Scientific Advisory Board of the United Nations [7] that “all countries, including the poorest, to invest at least 1% of their GDP on research and urged the most advanced countries to spend at least 3% of their GDP on research and development” (para. 51). Furthermore, it states that governments must approve “policies and regulations which foster scientific research, allocating appropriate resources in the budgets and, in general, creating an enabling and participatory environment for the conservation, development and diffusion of science and technology” (para. 50).

The General Comment on science and economic, social and cultural rights is an important addition to the human rights that are traditionally invoked in the global health arena. Most importantly, it reinforces the power of human rights law to frame the global health discourse not only in terms of public health necessity to act but also in terms of a legal duty to act. The global health community should not forget that ensuring good science and the attainment of the highest level of health are human rights and an indispensable dimension of global health policy. Only when cultivated and guaranteed as a human right, science provides the support needed to fight global health emergencies.
